# Combined Oral Contraceptives and Venous Thromboembolism: Review and Perspective to Mitigate the Risk

**DOI:** 10.3389/fendo.2021.769187

**Published:** 2021-12-09

**Authors:** Laure Morimont, Hélène Haguet, Jean-Michel Dogné, Ulysse Gaspard, Jonathan Douxfils

**Affiliations:** ^1^ Research Department, Qualiblood s.a., Namur, Belgium; ^2^ Faculty of Medicine, Department of Pharmacy, Namur Research Institute for Life Sciences (NARILIS), Namur Thrombosis and Hemostasis Center (NTHC), University of Namur, Namur, Belgium; ^3^ Department of Obstetrics and Gynecology, University of Liège, Liège, Belgium

**Keywords:** contraception, combined oral contraceptives, venous thromboembolism, activated protein C resistance, standard of care, risk factors, hemostasis

## Abstract

Many factors must be considered and discussed with women when initiating a contraceptive method and the risk of venous thromboembolism (VTE) is one of them. In this review, we discuss the numerous strategies that have been implemented to reduce the thrombotic risk associated with combined oral contraceptives (COCs) from their arrival on the market until today. Evidences suggesting that COCs were associated with an increased risk of VTE appeared rapidly after their marketing. Identified as the main contributor of this risk, the dosage of the estrogen, i.e., ethinylestradiol (EE), was significantly reduced. New progestins were also synthetized (e.g., desogestrel or gestodene) but their weak androgenic activity did not permit to counterbalance the effect of EE as did the initial progestins such as levonorgestrel. Numerous studies assessed the impact of estroprogestative combinations on hemostasis and demonstrated that women under COC suffered from resistance towards activated protein C (APC). Subsequently, the European Medicines Agency updated its guidelines on clinical investigation of steroid contraceptives in which they recommended to assess this biological marker. In 2009, estradiol-containing COCs were marketed and the use of this natural form of estrogen was found to exert a weaker effect on the synthesis of hepatic proteins compared to EE. In this year 2021, a novel COC based on a native estrogen, i.e., estetrol, will be introduced on the market. Associated with drospirenone, this preparation demonstrated minor effects on coagulation proteins as compared with other drospirenone-containing COCs. At the present time, the standard of care when starting a contraception, consists of identifying the presence of hereditary thrombophilia solely on the basis of familial history of VTE. This strategy has however been reported as poorly predictive of hereditary thrombophilia. One rationale and affordable perspective which has already been considered in the past could be the implementation of a baseline screening of the prothrombotic state to provide health care professionals with objective data to support the prescription of the more appropriate contraceptive method. While this strategy was judged too expensive due to limited laboratory solutions, the endogenous thrombin potential-based APC resistance assay could now represent an interesting alternative.

## Manuscript

When oral contraceptives became generally available during the early 1960s, their use rapidly increased and in 2019, it was estimated that over 150 million women were using the pill worldwide (1). With such a large number of subjects on this medication, and with a majority using combined estrogen-progestin products, even a small increase in risk of serious side effects affects the lives of many women. Moreover, as contraceptive therapies are administered to healthy young women with the aim to prevent unwanted pregnancies, the occurrence of side effects should be as low as possible and risk minimization strategies should be implemented accordingly.

### Introduction: The History of Combined Oral Contraceptives

**Figure 1:**
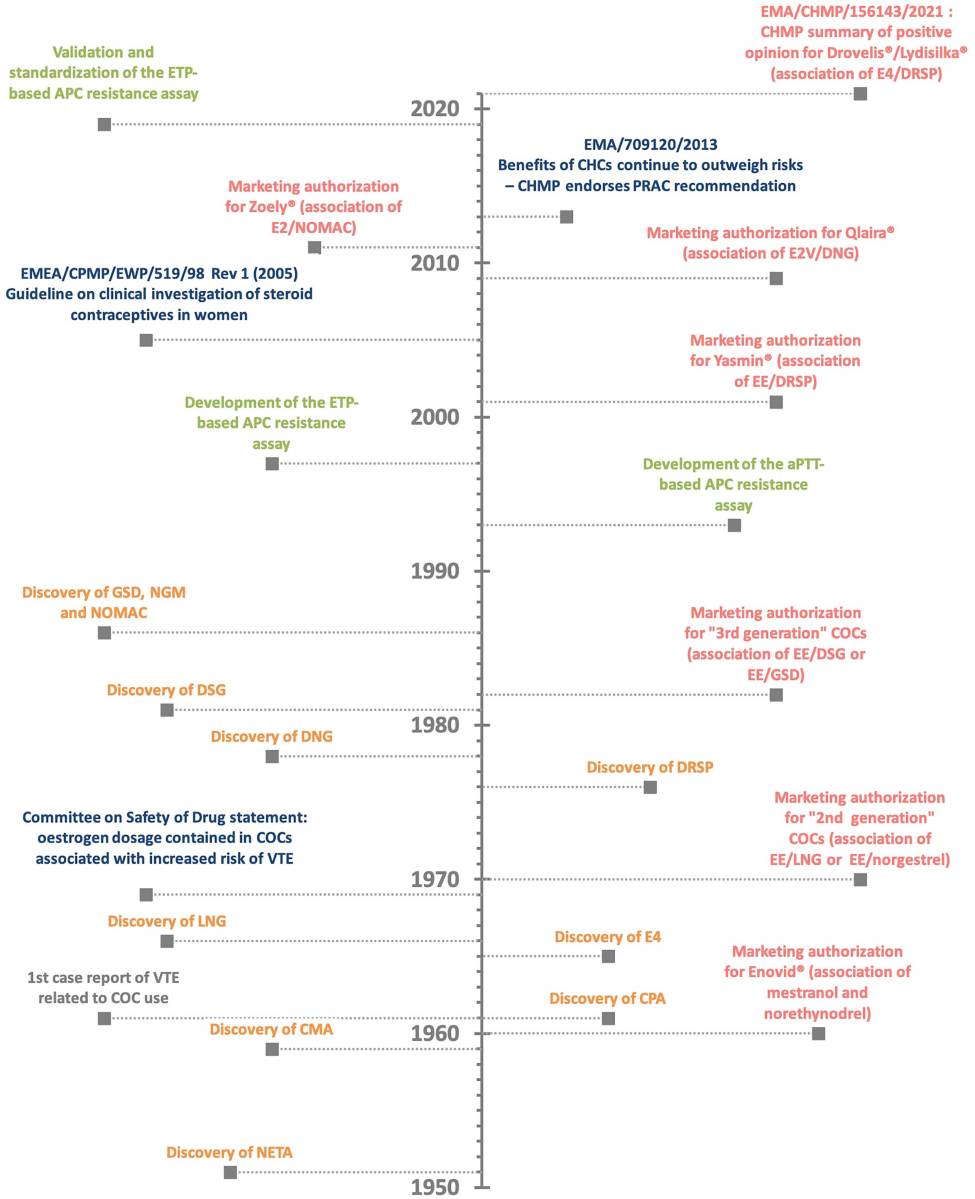
The history of combined oral contraceptive (COC) and the related risk of venous thromboembolism (VTE). Discovery of estrogen and progestin compound (in orange) - marketing authorization for combined oral contraceptives (in red) – authorities’ statement (in blue) – assay development (in green). APC, activated protein C resistance; aPTT, activated partial thromboplastin time; CHMP, Committee for Medicinal Products for Human Use; CHC, combined hormonal contraceptives; CMA, chlormadinone acetate; COC, combined oral contraceptives; CPA, cyproterone acetate; DNG, dienogest; DRSP, drospirenone; DSG, desogestrel; EE, ethinylestradiol; EMA, European Medicines Agency; ETP, endogenous thrombin potential; E2, 17β-estradiol; E2V, estradiol valerate; E4, estetrol; GSD, gestodene; LNG, levonorgestrel; NETA, norethisterone acetate; NGM, norgestimate; NOMAC, nomegestrol acetate; PRAC, Pharmacovigilance Risk Assessment Committee; VTE, venous thromboembolism event.

**Figure 2:**
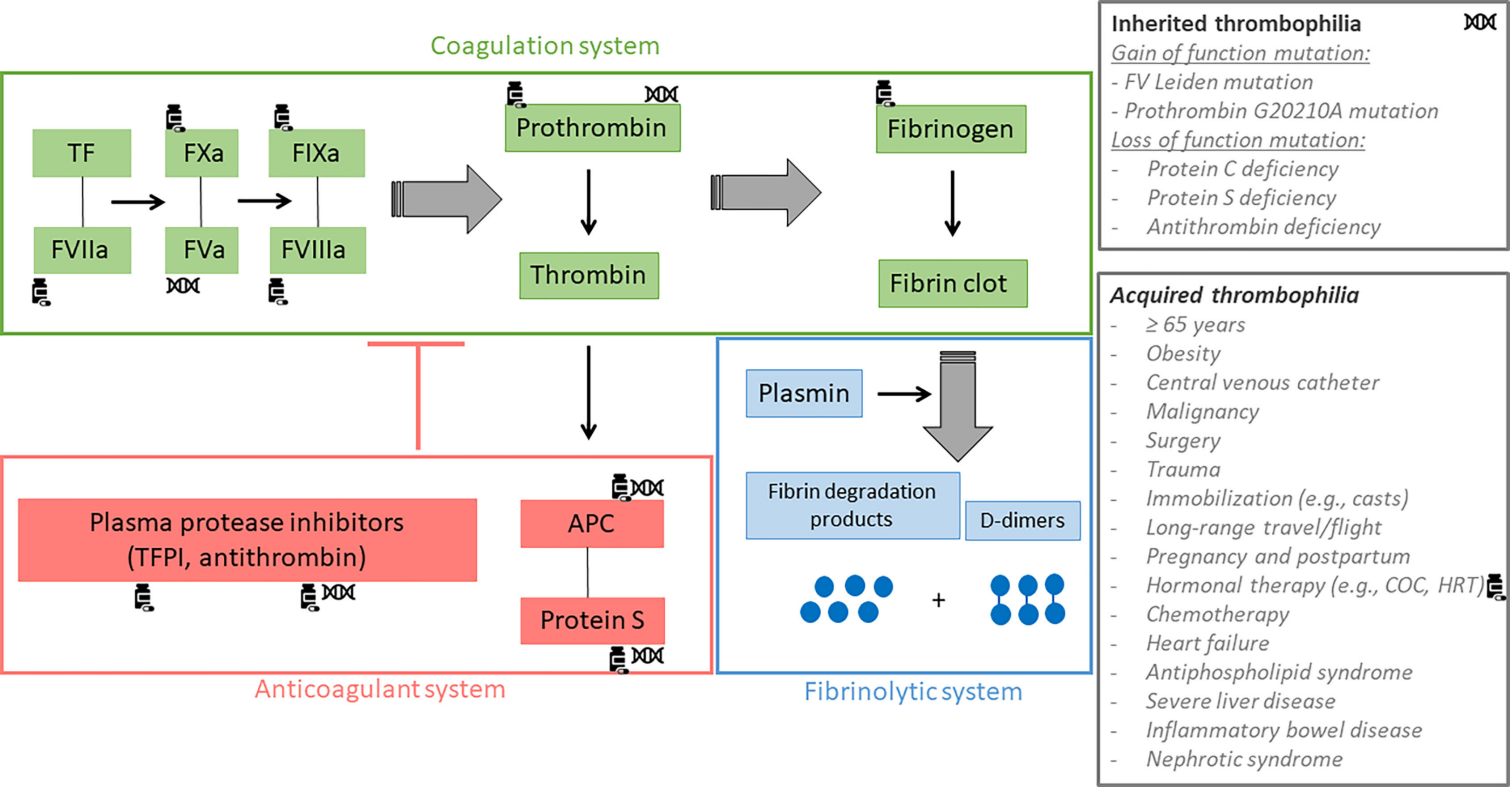
Coagulation, fibrinolytic and anticoagulant systems. The green rectangle refers to the coagulation system, the blue rectangle refers to the fibrinolytic system and the red rectangle refers to the anticoagulant system. Tissue factor (TF) plays an important role in the initiation of the coagulation process. Combined with activated factor VII, the complex activates FIX and FX. Subsequently, FIXa and FXa form two complexes with activated FV and activated FVIII respectively, leading to the conversion of prothrombin into thrombin. Once formed, thrombin cleaves fibrinogen to form the fibrin clot which is then degraded by the fibrinolytic system whose the main effector is plasmin. This releases fibrin degradation products and D-dimers. Thrombin also activates the protein C system in order to down regulate its own production. Indeed, once activated by thrombin, protein C forms a complex with protein S in order to inactivate factor Va and factor VIIIa, the two main co-factors of the intrinsic (IXa-VIIIa) and the prothrombinase (Xa-Va) complexes. The generation of thrombin is also regulated by other protease inhibitors like antithrombin (AT) and tissue factor pathway inhibitor, widely known as TFPI. Inherited and acquired thrombophilia can disrupt the coagulation, the fibrinolytic or the anticoagulant system leading to an increased risk of venous thromboembolism. Coagulation factors impacted by inherited thrombophilia are marked with the DNA-symbol and those impacted by the intake of combined oral contraceptives (COC) are marked with the drug-symbol. APC, activated protein C; TF, tissue factor; TFPI, tissue factor pathway inhibitor.

Evidence suggesting that oral contraceptives were associated with an increased risk of venous thromboembolism events (VTE) appeared rapidly after they were marketed **(** **Figure 1** **)**. The first case of VTE was reported in 1961 in a 40-year-old woman who had been given Enovid^®^, an association of 150 µg of mestranol and 10 mg of norethynodrel for the control of endometriosis (2, 3). This association of an estrogen with a progestogen marked the beginning of the combined hormonal contraceptives (CHCs) era which were initially only given by the oral route. In the subsequent years, it was reported that the occurrence of VTE was higher with combined oral contraceptives (COCs) containing more than 50 µg of estrogen, either mestranol or ethinylestradiol (EE), compared to preparations containing a lower dosage (4). Following the publication of these reports, the British Committee on Safety of Drugs, one of the leading instance for drug safety at that period, issued, in 1969, a statement indicating that the dose of estrogen contained in oral contraceptives was positively associated with an increased risk of VTE (5). The dosage of estrogen was then reduced from 75 µg, or more, to 50 µg and afterwards to 30 and 20 µg. At the present time, some COCs even contain 10 µg of EE (e.g., Lo-Loestrin^®^ Fe) (6). Comparisons of COCs containing 50 and 30 µg of EE on blood coagulation, fibrinolysis and platelets confirmed the estrogen dose-dependent effect. Enhanced platelet activity, increased levels of factor II, VII, VIII, IX and X, fibrinogen and soluble fibrin and decreased levels of antithrombin (AT) and vessel wall fibrinolytic activator were observed with both preparations, but they were less pronounced with 30 µg COC preparations (3, 6–8) **(** **Figure 2** **)**.

The progestogen compound found in oral contraceptives also changed over time with several pharmacomodulations aiming at providing different estrogenic, androgenic, glucocorticoid or mineralocorticoid profiles (9). Indeed, endogenous progesterone, synthetized in the ovarian corpus luteum, possesses antiestrogenic, antiandrogenic and antimineralocorticoid activities. Thus, synthetic progestogens used for contraception also mimicked some of these properties (10). Overall, these compounds are characterized by a 4-ring steroid skeleton and are classified based on their structure (**Table 1**). The first progestogens, synthetized in 1951, were norethynodrel and norethisterone acetate (also known as norethindrone acetate). They are characterized by a 19-nortestosterone structure and are regarded as estranes (carbon-18). Shortly afterwards, in 1959, 17-hydroxyprogesterone derivatives categorized as pregnanes (carbon-21) were also synthetized. These included chlormadinone acetate and cyproterone acetate. In 1966, other 19-nortestosterone derivatives were discovered with norgestrel and levonorgestrel but differed from norethynodrel and norethisterone acetate as they possess a 17-carbon structure. They are better known as gonanes. During the 1980s, three new progestogens, derived from levonorgestrel, were developed, that are desogestrel, gestodene and norgestimate. Drospirenone, dienogest and nomegestrol acetate are, for their part, considered to be the new progestogens. They were designed to bind more specifically to the progestogen receptors and to a lesser extent to the other steroid receptors in order to reduce undesirable effects. Regarding their structure, dienogest is a gonane derivative, nomegestrol acetate is a norpregnane (carbon-20) derivative and drospirenone is unique in its category and derivates from spironolactone (11–15) (**Table 1** **).** Combined oral contraceptives were usually classified into three generations, related to their arrival on the market. First generation contained high doses of EE (50 µg or more) associated with norethynodrel and with norethisterone acetate. They are no longer used in COC preparations. Second and third generations contain a lower dose of EE (20 or 30 µg). The associated progestogen is levonorgestrel in 2^nd^ generation COCs and desogestrel, gestodene or norgestimate in 3^rd^ generation COCs. Actually, this nomenclature was introduced by the pharmaceutical companies with the aim of boosting sales since the idea of a “new” generation suggests improvements and better efficacy and/or safety profile, the two latter being not supported by epidemiological data (16). This misleading classification led to inconsistencies like norgestimate-contained COC, categorized as 3^rd^ generation, which is in fact a prodrug of levonorgestrel and its 3-oxime metabolite, renamed norelgestromin by the pharmaceutical company (17). Although it is tempting to turn then to a chemical classification, this is not the Holy Grail since among compounds with nearly similar chemical structure, e.g. levonorgestrel and gestodene, the pharmacodynamic action when binding to the different steroid receptors may differ (18). Therefore, a pharmacodynamic classification should be preferred considering the activities of the progestogen when associated with an estrogen, the potency of the latter being determinant for the total estrogenicity of the association.

**Table 1:** Progestogens - Discovery, chemical structure, androgenic activity, associated estrogen compound in combined oral contraceptives (COC) and classification under generation.

Progestogen	Short name	Discovery date	Chemical structure	Androgenic activity	Anti-androgenic activity	Associated estrogen compound in COC	Classification under generation
**Norethisterone acetate**	NETA	1951	Estrane (18-carbon structure) 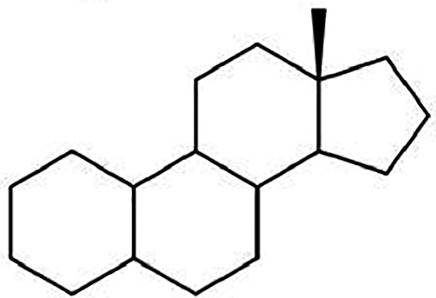	+	–	EE ≥ 50 µg	1^st^ generation COC
**Norethynodrel**	/	1957		+	–		
**Lynestrenol**	/	1961		+	–		
**Levonorgestrel**	LNG	1966	Gonane (17-carbon structure) 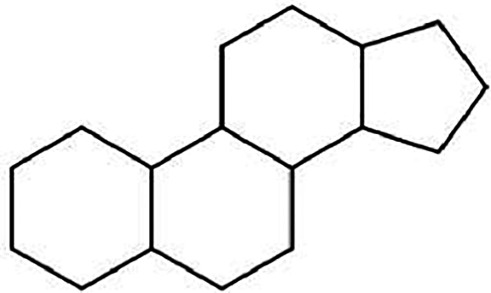	++	–	EE 20 µg/30 µg	2^nd^ generation COC
**Norgestrel**	/	1966		++	–		
**Desogestrel**	DSG	1981		+	–	EE 20 µg/30 µg	3^rd^ generation COC
**Gestodene**	GSD	1986		+	–		
**Norgestimate**	NGM	1986		+	–		
**Chlormadinone acetate**	CMA	1959	Pregnane (20-carbon structure) 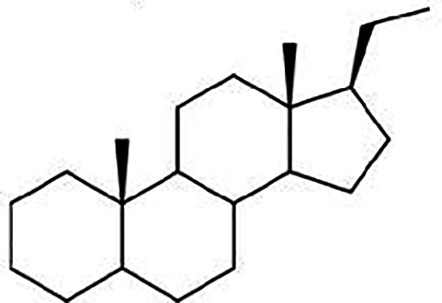	–	++	EE 30 µg	Unclassified/other
**Cyproterone acetate**	CPA	1961		–	++	EE 35 µg	
**Drospirenone**	DRSP	1976	Spironolactone derivative	–	+	EE 20 µg/30 µg	
**Dienogest**	DNG	1978	Gonane (17-cabon structure)	–	+	EE 30 µg E2V 1-3mg	
**Nomegestrol acetate**	NOMAC	1975	Norpregnane (20-carbon structure) 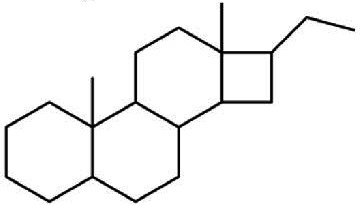	–	+	Micronized E2 1.5 mg	

EE, ethinylestradiol; E2, estradiol.

## Discussion

### Third Generation and New Oral Contraceptives Controversy

Going back into the 80s, the main reason that led to the development of new progestogens was related to the side effects induced by the combination of EE with levonorgestrel or norethisterone acetate, such as acne, hirsutism and weight gain (19). The decreased androgenic activity of desogestrel, gestodene and norgestimate has certainly permitted to reduce these adverse effects. However, an increased risk of VTE was observed compared to levonorgestrel as reported by 4 independent epidemiological studies in 1995-1996 (20–23). Although these results had been debated due to possible impact of confounding factors and bias, such as healthy user bias, introduction bias, duration of oral contraceptive use, COC switching, prescribing bias, diagnosis suspicion and referral bias or the source of funding, the increased risk with these progestogens has been subsequently confirmed by other investigations (24–29). As earlier studies suggested that the increased risk of VTE associated with COCs was only estrogen dose-dependent, the most plausible explanations at that time were these biases. Nevertheless, the meta-analysis of Kemmeren et al. published in 2001, revealed that even after stratifying for various factors like first time user; age, (i.e. younger and older than 25 years); duration of use, (i.e. less or more than 1 year); confirmation status of VTE cases and source of funding, (i.e. industry or non-industry sponsored study); the risk remained more elevated with the so-called 3^rd^ generation COCs compared to the 2^nd^ generation COCs (30).

This phenomenon was also observed with COCs containing EE associated with drospirenone and cyproterone acetate. Unlike levonorgestrel, desogestrel, gestodene and norgestimate, the latter two progestins are completely devoid of any androgenic or glucocorticoid effects (31). Cyproterone acetate possesses even the highest antiandrogenic activity which makes this molecule the ideal candidate to treat severe acne and hirsutism in women (18). Drospirenone, on the other hand, differs from the other progestogen by its chemical structure derived from spironolactone conferring an antimineralocorticoid activity. This allows to offset the fluid retention induced by estrogens and prevent weight gain during COC therapy (32). The development of these two progestogens with antiandrogenic and antimineralocorticoid properties provided us with compounds closer to progesterone permitting the reduction of the above-mentioned adverse effects but on the flip side of the coin, they clearly led to an increased risk of VTE when associated with EE (33, 34).

**Figure 3:**
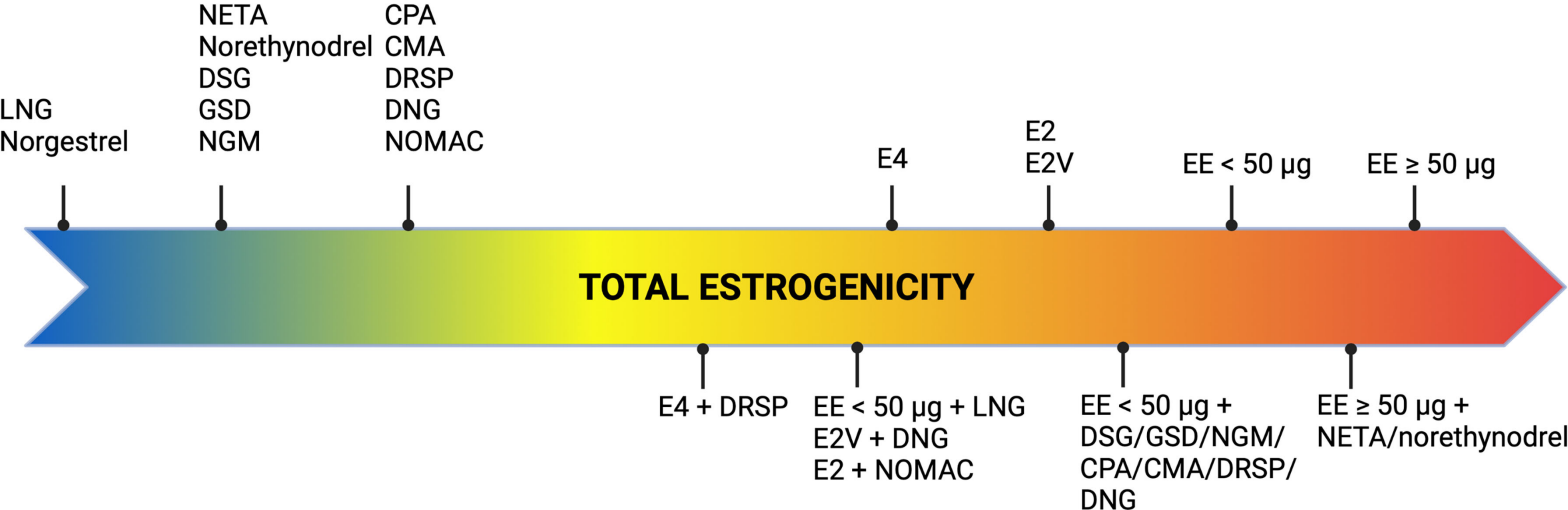
Concept of estrogenicity with combined oral contraceptives. CMA, chlormadinone acetate; CPA, cyproterone acetate; DNG, dienogest; DRSP, drospirenone; DSG, desogestrel; EE, ethinylestradiol; E2, estradiol; E2V, estradiol valerate; E4, estetrol; GSD, gestodene; LNG, levonorgestrel; NETA, norethisterone acetate; NGM, norgestimate; NOMAC, nomegestrol acetate.

As evidence demonstrated that COCs with the same estrogen dose but different progestogens were associated with differential VTE risk, it was suggested that the progestogen compound might play a role in thrombosis development. However, as progestin only contraceptives do not interfere with coagulation protein synthesis, the difference in VTE risk specific to each COC could only be attributed to a distinctive modulation of the procoagulant effect of EE, exerted by the progestogens (35, 36). This modulation is actually related to the activity of the progestogen compound on hormonal receptors and especially on androgen receptors (37). Levonorgestrel, characterized by a strong androgenic activity, balances out to a certain degree the estrogen-dependent alteration in hemostasis and hepatic protein synthesis. Consequently, levonorgestrel further offsets the procoagulant effect induced by EE compared to desogestrel, gestodene, norgestimate, drospirenone and cyproterone acetate, which have a weaker androgenic or even an antiandrogenic activity (11, 14, 18). An interaction between androgen receptors and estrogens responsive elements could be a hypothesis to explain this phenomenon (38). It could prevent the activation of target genes coding for hepatic proteins such as coagulation factors, thus modulating the effect of EE (38). Ultimately, the estrogenicity of a COC is the sum of both the estrogen and the progestogen contribution and excessive estrogenicity was reported to increase the risk of VTE (**Figure 3**) (39). The biomarker that best reflects the estrogenicity is the sex hormone binding globulin (SHBG), a carrier protein for estrogen and testosterone, produced by the liver, and whose synthesis is highly estrogen sensitive (39). The oral intake of EE alone leads to a significant dose-dependent increase in SHBG whereas progestogens induce a decrease of SHBG, the extent being dependent of its androgenic activity (39). Thus, progestogens with a potent androgenic activity cause a more pronounced reduction in SHBG levels than less androgenic or antiandrogenic ones. As a consequence, the so-called 3^rd^ generation COCs and those containing EE combined with drospirenone or cyproterone acetate induce a drastic increase in SHBG levels compared to the so-called 2^nd^ generation COCs (39–41).

As a relationship has been observed between SHBG levels and the increased risk of VTE associated with COC use, the assessment of this liver protein represents an important biomarker to consider during the development of steroid contraceptives (42, 43). However, SHBG is not a coagulation protein and other biological variables better reflecting the impact of COCs on hemostasis would seem more appropriate to reflect the potential induced prothrombotic switch (44).

### Acquired Activated Protein C Resistance With Oral Contraceptives: A Key Factor in Venous Thromboembolism Risk

In the 1990s, it was estimated that VTE affected 1 per 1,000 people annually, and that family history of thrombosis was often found in these patients (45). However, the main inherited thrombophilia investigated at that time such as deficiency of antithrombin, protein C and protein S accounted for only 5- to 10% of the cases (45). This suggested that other genetic defects predisposed to the development of VTE and had yet to be identified. In 1993, Dahlbäck et al. started to investigate the protein C - protein S anticoagulant pathway (46). They developed an activated partial thromboplastin time (aPTT)-based method to determine the sensitivity of a patient’s plasma towards exogenous activated protein C (APC). The aPTT assay is based on the principle that in citrated plasma, the addition of phospholipids, activator of factor XII (e.g., silica) and calcium chloride allow for formation of a stable clot. The time from activation to formation of a stable clot is recorded in seconds, and represents the aPTT (47). After the addition of APC, they noted that plasma from patients with thrombosis and family history of thrombosis had a shorter prolongation of the aPTT clotting time compared with plasma from healthy individuals (46). Therefore, as APC is expected to delay the aPTT in normal plasma, due to its ability to inactivate factor Va and VIIIa, the observed phenomenon was defined as APC resistance. To ease the expression of results, an APC sensitivity ratio, defined as the aPTT (+APC) divided by the aPTT (-APC), was calculated and is depicted in equation 1:


(Equation 1) $$\text{APCsr}(\text{aPTT})=\frac{sample\;aPTT(+APC)}{sample\;aPTT(-APC)}$$

The more resistant a sample is to APC, the lower the numerator (+APC condition) compared to the denominator (-APC condition) and therefore the closer the ratio is to zero (48). These findings were confirmed by others (49, 50) and the phenotype of APC resistance was latter associated with a mutation in the coding region of factor V, better known as Factor V Leiden mutation (51). This mutation abolishes one of the APC-cleavage sites on FVa which leads to a slowdown inactivation of FVa. This further prevents FVa to be converted into a functional APC cofactor needed for FVIIIa inactivation and, as a consequence, the inactivation of both FVa and FVIIIa are delayed (52). The discovery of this genetic mutation indirectly permitted to better understand the etiology of COC-induced VTE events. Indeed, many patients were found to have a resistance to APC without carrying a FV Leiden mutation, suggesting either the existence of other genetic mutations or the existence of acquired factors capable of interfering with the inhibitory activity of APC (51). In the study of Koster et al. healthy men had a more pronounced anticoagulant response to APC than women, suggesting a possible influence of female sexual hormones (49). It was then assumed that a poor response to APC could explain, at least in part, the procoagulant state observed in users of oral contraceptives. This was confirmed in 1995 by Henkens et al. and Olivieri et al. who demonstrated that COC therapy could induce acquired APC resistance independently from genetic mutation of the factor V Leiden (53, 54). Although these results were tremendous and important to support the observed increased risk of VTE with COCs and conditions leading to APC resistance, other tests which account for the entire coagulation process were developed.

**Figure 4:**
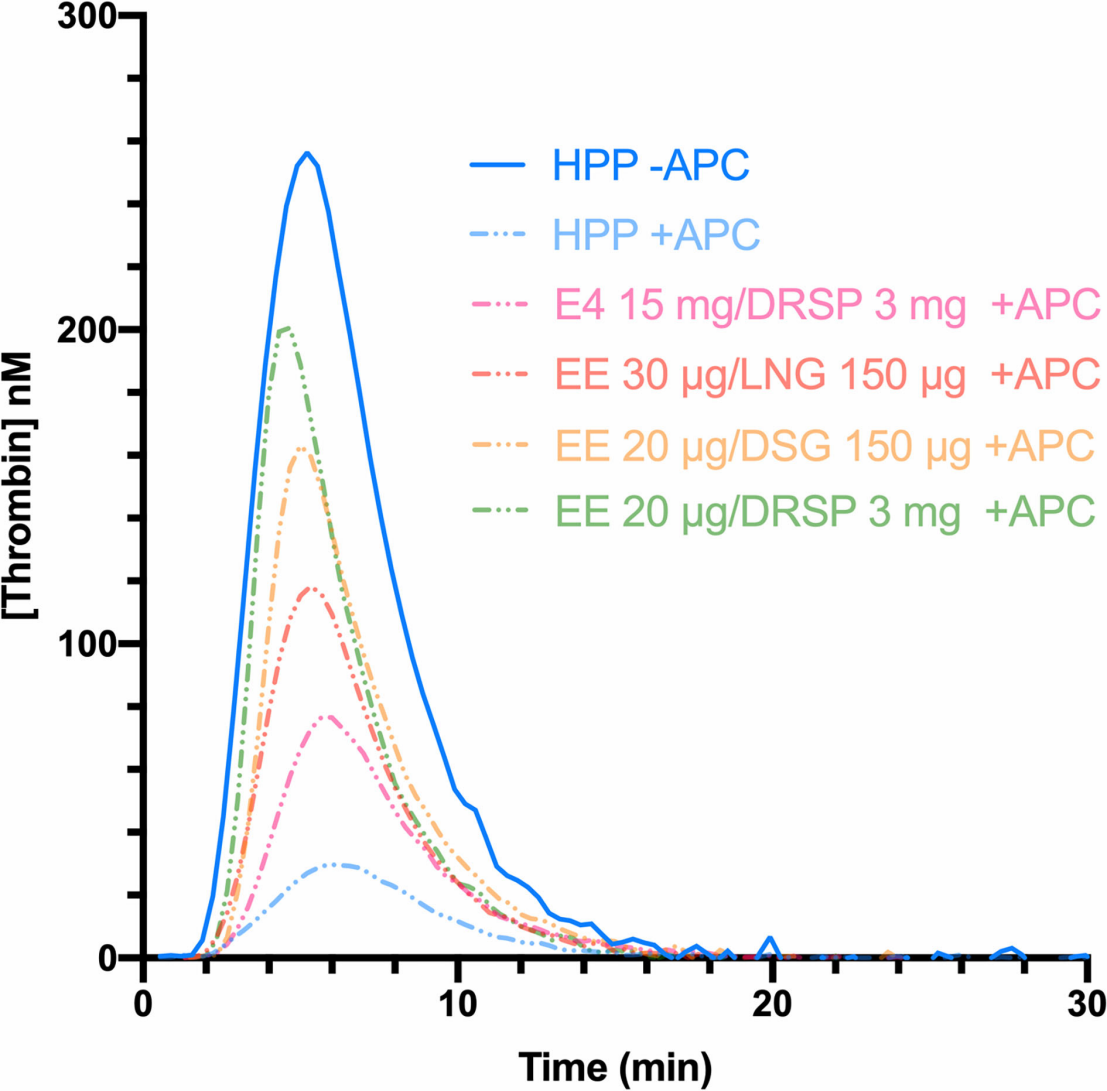
Thrombin generation curves in absence (continuous line) and in presence of APC (dotted lines) of a healthy pooled plasma (blue) and women using combined oral contraceptives containing either ethinylestradiol (EE) with drospirenone (green), EE with desogestrel (yellow), EE with levonorgestrel (red), or estetrol with drospirenone (pink). The area under the curve represents the endogenous thrombin potential (ETP) parameter. In presence of APC, the ETP is higher with the use of combined oral contraceptives (e.g., drospirenone or desogestrel) compared to the healthy pooled plasma (composed of men and women not using hormonal contraception), leading to a resistance towards the APC. APC, activated protein C; DRSP, drospirenone; DSG, desogestrel; EE, ethinylestradiol; E4, estetrol; HPP, healthy pooled plasma; LNG, levonorgestrel.

In 1997, Nicolaes et al. reported on a new method based on the continuous measurement of thrombin generation over time, in the presence and absence of exogenous APC (55). Thrombin generation assay (TGA) is based on the potential of a plasma to generate thrombin over time, after activation of coagulation by addition of phospholipids, tissue factor and calcium. The resulting thrombin generation curve reflects all the pro- and -anticoagulant reactions that regulate both thrombin formation and inhibition. In contrast to the aPTT assay, which only assesses the initiation phase of the coagulation, TGA is a global assay investigating the initiation, the propagation and the termination phase of the coagulation. The addition of APC induces a lowering of thrombin generation which is quantitated by the endogenous thrombin potential (ETP), corresponding to the area under the thrombin generation curve **(** **Figure 4** **)**. This test was therefore referred as the ETP-based APC resistance assay and results were expressed as normalized APC sensitivity ratio (nAPCsr). This unit corresponds to the ratio of the ETP measured in presence and absence of APC divided by the same ratio determined in a reference plasma (Equation 2).


(Equation 2) nAPCsr=Sample plasma ETP(+APC)Sample plasma ETP(−APC)Reference plasma ETP(+APC)Reference plasma ETP(−APC)

The obtained ratio stands between 0 and 10 and conversely to the aPTT-based assay, the higher the nAPCsr, the more resistant the patient is to APC. A comparison between both tests is shown in **Table 2**.

**Table 2:** Assessment of acquired activated protein C resistance.

***Assay***	***aPTT-based APC resistance assay***	***ETP-based APC resistance assay***
**Development year**	1993	1997
**Principle**	Relies on the ability of APC to prolong the aPTT, *via* its anticoagulant properties	Relies on the ability of APC to reduce thrombin generation, *via* its anticoagulant properties
**Trigger**	*Via* intrinsic pathway	*Via* extrinsic pathway
**End-point**	Clotting time (initiation phase)	Endogenous thrombin potential (initiation, propagation and termination phase)
**Results**	$\text{APCsr}=\frac{sample\;aPTT(+APC)}{sample\;aPTT(-APC)}$	nAPCsr=Sample plasma ETP(+APC)Sample plasma ETP(−APC)Ref plasma ETP(+APC)Ref plasma ETP(−APC)
**Interpretation**	Low APCsr indicates failure of APC to prolong the clotting time of plasma, defined as APC resistance	High nAPCsr indicates impaired down-regulation of thrombin generation by APC, defined as APC resistance
**Main determinants**	FV Leiden FV levels FVIII levels Prothrombin levels	FV Leiden FV levels TFPI levels PS levels

APC, activated protein C; APCsr, activated protein C sensitivity ratio; aPTT, activated partial thromboplastin time, ETP, endogenous thrombin potential; FV, factor V; FVIII, factor FVIII; nAPCsr, normalized activated protein C sensitivity ratio; PS, protein S; Ref plasma, reference plasma; TFPI, tissue factor pathway inhibitor. Genetic risk factors, such a Factor V Leiden mutation and acquired risk factors, such as the use of oral contraceptive can interfere with the expression of the APC anticoagulant activity. The phenomenon is defined as APC resistance and can be assessed by two functional tests, the activated partial thromboplastin time (aPTT)-based and the endogenous thrombin potential (ETP)-based APC resistance assays.

**Figure 5:**
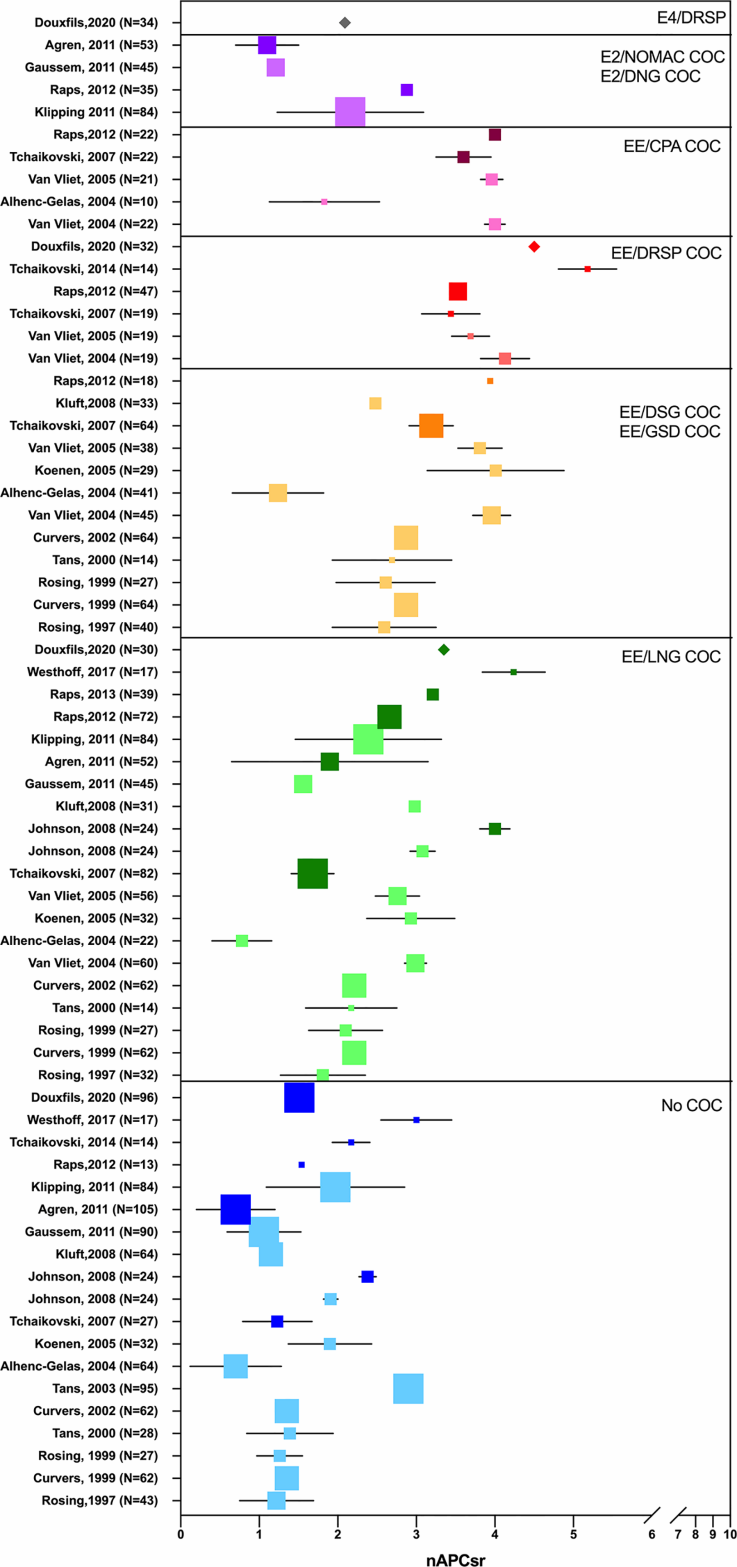
Synthesis of studies from 1997 to 2020 investigating the impact of oral contraceptives on the activated protein C (APC) resistance, when expressed as normalized APC sensitivity ratio (nAPCsr). The X-axis represents nAPCsr in absolute values and the Y-axis represents the studies included (first author, year, sample size). Studies are cited multiple times depending on the investigated combined oral contraceptives. Blue squares represent nAPCsr values (± SD) for “no-COC user” group; green squares represents nAPCsr values (± SD) for “EE-LNG user” group; orange squares represents nAPCsr values (± SD) for “EE-DSG and EE-GSD user” group; red squares represents nAPCsr values (± SD) for “EE-DRSP user” group; pink squares represents nAPCsr values (± SD) for “EE-CPA user” group; purple squares represents nAPCsr values (± SD) for “E2/NOMAC or E2/DNG user” group and the grey square represents nAPCsr value (± SD) for “E4/DRSP user” group. The size of the square is related to the sample size. The clearest square are chromogenic-based ETP-based APC resistance studies and the darkest ones are calibrated automated thrombogram-based studies. The validated and standardized ETP-based APC resistance assay is represented by a diamond. APC, activated protein C; COC, combined oral contraceptive; CPA, cyproterone acetate; DSG, desogestrel; DNG, dienogest; DSPR, drospirenone; EE, ethinylestradiol; E2, estradiol; GSD, gestodene; LNG, levonorgestrel; nAPCsr, normalized activated protein C sensitivity ratio; NOMAC, nomegestrol acetate; SD, standard deviation.

Depending on the test used, i.e., aPTT-based or ETP-based APC resistance assay, the differences observed between non-users and COC users were not the same. The ETP-based APC resistance assay revealed to be more sensitive to COC impact on hemostasis and significant differences were observed between COC preparations (e.g., levonorgestrel-containing product versus desogestrel- or gestodene-containing product) **(** **Figure 4** **)** (56, 57). Indeed, these two assays are not sensitive to the same factors: the aPTT-based assay is more sensitive towards levels of prothrombin and FVIII while the ETP-based assay is most influenced by free tissue factor pathway inhibitor (TFPI) and free protein S levels (58). As these latter factors are much more impacted by COCs than the two formers, it may in part explain the inconsistent results between these two functional APC resistance assays (59, 60). Following the widespread use of this biomarker to evaluate the increased risk of VTE associated with COCs in the early 2000s, the Committee for Medicinal Products for Human Use (CHMP) of the European Medicines Agency (EMA) stated, in 2005, that APC resistance should be investigated during the development of new steroid contraceptives (42). Nevertheless, the lack of standardization of this method has remained a problem for many years, and this has been reflected in highly variable results from one study to another, as depicted in **Figure 5**. Recently, Douxfils et al. developed and validated a new ETP-based APC resistance method aiming to provide a harmonized scale of nAPCsr. This permits to reduce the inter-laboratory variability and allowed lab-to-lab and study-to-study comparison and evaluation (61). In addition, the typical information obtained by thrombin generation investigation is available, providing much more information than the APC resistance itself. As it enables the assessment of the global coagulation process, this assay is also sensitive towards other factors of thrombogenicity like the prothrombin G20210A mutation, antithrombin and protein S deficiencies or FVIII levels (62–65). The ETP-based APC resistance assay may thus provide information on hemostatic functions which are linked to an increased risk of thrombosis to help the prescriber in clinical decision making.

### Towards a Safer Alternative With Estradiol-Based COC

The estrogen component remained unchanged for a long time, with the majority of COCs containing EE. Nevertheless, to further improve the tolerability of COCs and to broaden the choice for COC users, attempts have been made to replace EE with natural estradiol (E2) (66, 67).

The contraceptive efficacy of COCs is primarily derived from the action of the progestogen compound but the estrogenic moiety is also an important contributor of the efficacy of COCs. Indeed, estrogens are partly responsible for suppressing the follicle-stimulating hormone, they potentiate the activity of the progestin component, by increasing progestin receptor concentration and they stabilize the endometrium so that irregular and unwanted bleeding can be minimized (68, 69). Although these desired effects on the reproductive organs, the occurrence of thrombotic events for which estrogen was held responsible, led to a drastic reduction in EE dosage, as stated above. However, reducing the amount of estrogen, up to 10 µg of EE, with the aim to improve safety resulted in unacceptable changes in bleeding patterns compared to higher doses (69). Therefore, the development of COCs containing natural estrogen, i.e., E2 was then suggested as an alternative of EE (69). The interest of using E2, as the estrogen component in COCs was raised in the 1970s with the aim of improving the tolerability. Moreover, several studies showed that E2 impacted to a lesser extent the synthesis of hepatic protein compared to EE (70–72).

Because of its low oral bioavailability, E2 is either in a micronized form or esterified. Estradiol valerate (E2V) presents similar pharmacokinetic and pharmacodynamic characteristics to that of E2 as it is rapidly converted to E2 in the intestines and the liver (1 mg of E2V yields 0.75 mg E2) (73). Indeed, the cleavage of E2V to E2 and the valeric acid occurs during absorption by the intestinal mucosa and in the course of the first pass effect. This gives rise to E2 and its metabolites estrone (E1) and estriol (E3) (69).

First E2-containing COCs, introduced in the 1980s, were monophasic preparations containing between 1 and 3 mg of micronized E2. Although these preparations demonstrated effective ovulation inhibition and provided excellent contraceptive efficacy, these benefits were outweighed by unacceptable bleeding irregularities (69). An inappropriate estrogen-progestogen ratio or a suboptimal E2 dosage was pointed out as the plausible explanation for this failure in cycle control (67, 74). To address this issue, a preparation was developed in which E2V was combined with dienogest, in a four-phasic dosing regimen incorporating an estrogen step-down and a progestin step-up over 26 days (66, 75). The treatment consists of the administration of E2V 3 mg for 2 days – E2V 2 mg/dienogest 2 mg for 5 days - E2V 2 mg/dienogest 3 mg for 17 days - E2V 1 mg for 2 days - and placebo for 2 days (66). This dynamic dosing regimen was designed to ensure estrogen dominance in the first part of the cycle and progestin dominance in the mid to late part of the cycle, thereby optimizing the control of bleeding (76). Ahrendt et al. demonstrated that this regimen certainly improved the cycle control and appeared to be associated with shorter and lighter bleeding compared with EE/levonorgestrel (67). Furthermore, unlike other progestogens, dienogest is a 19 nor-testosterone derivative, with a 17-cyanomethyl instead of an ethinyl group at the C-17 position (77) and possesses therefore a strong affinity for progesterone receptors, displays an antiandrogenic activity and lacks estrogenic, glucocorticoid and androgenic properties. This suggested that this progestogen would exert only minor metabolic effects (78). Studies of Junge et al. and Klipping et al. confirmed the minimal impact of this preparation on lipid, hemostasis and carbohydrate metabolism (75, 78). This E2-containing OC became globally available in 2009 under the trade name Qlaira^®^. A few years later, in 2011, a new monophasic pill containing 1.5 mg of micronized 17β-estradiol and 2.5 mg nomegestrol acetate was introduced in the European market, under the tradename Zoely^®^, and consisted of a 24- days regimen followed by a 4-day placebo (79). Nomegestrol acetate is a progesterone derivative, and more specifically, a 19-norpregnane, possessing an antiestrogenic activity on the endometrium and a moderate antiandrogenic activity. Through this enhanced selectivity profile, this preparation was expected to provide acceptable cycle control and limit cardiovascular and metabolic side effects. This was confirmed by Agren et al. and Gaussem et al. who revealed that E2/nomegestrol acetate had a similar safety and tolerability profile to EE/levonorgestrel (80, 81). As these studies were based on biological and pharmacological data with no attempts to correlate hemostatic changes with VTE risk, epidemiological data on the risk of VTE associated with these E2-containing COCs were requested by the regulatory agencies. Two large international active surveillance studies, the INAS-SCORE (NCT01009684) and the PRO-E2 (NCT01650168) were initiated to assess the risk of short and long-term use of E2V/dienogest and E2/nomegestrol acetate respectively (82, 83). In comparison with COC-levonorgestrel users, the incidence of VTE was slightly lower in users of E2V/dienogest and E2/nomegestrol acetate (**Table 3**). This suggests that EE/levonorgestrel is not the only option for minimizing the risk of VTE associated with COC use, but estradiol-containing product is equally safe (86). This also reflects that those biological investigations may, at least in part, correlate with epidemiological data. This also permit to put the pharmacodynamics data requested by the EMA during the development of steroid contraceptives into a more clinical context.

**Table 3:** Estimated risk of venous thromboembolism events (VTE) with combined oral contraceptives (COC).

Risk group	Estimated risk of VTE	Reference
**Non-pregnant non-user**	2/10 000 WY	EMA/739865/2013 (84)
**Pregnant and postpartum women**	20/10 000 WY	30-year population-based study (85)
**User of COC containing** **- EE + LNG** **- EE + NETA** **- EE + NGM**	5-7/10 000 WY	EMA/739865/2013 (84)
**User of COC containing** **- EE + GSD** **- EE+ DSG** **- EE+ DRSP**	9-12/10 000 WY	EMA/739865/2013 (84)
**User of COC containing** **- EE + CMA**	Unknown	N/A
**User of COC containing** **- EE + DNG**	8-11/10 000 WY (update from the EMA)	EMA
**User of COC containing** **- E2V + DNG**	7.0/10 000 WY versus 3.5 in non-user and 9.9 in LNG/EE users	INAS-SCORE study (82)
**User of COC containing** **- E2/NOMAC**	2.0/10 000 WY versus 1.8 in non-user and 3.0 in LNG/EE users	PRO-E2 (83)
**User of COC containing** **- E4/DRSP**	Pending	PASS required by the EMA and FDA

CMA, chlormadinone acetate; COC, combined oral contraceptives; DNG, dienogest; DRSP, drospirenone; DSG, desogestrel; EE, ethinylestradiol; EMA, European Medicines Agency; EURAS, European Actie Surveillance Study; E2, 17β-estradiol; E2V, estradiol valerate; E4, estetrol; GSD, gestodene; INAS-CELINA, International Active Surveillance Study: Choice of Estrogen and Long-term Investigation of Nomegestrol Acetate; INAS-SCORE, International Active Surveillance study “Safety of Contraceptives: Role of Estrogens”; LNG, levonorgestrel; NETA, norethisterone acetate; NGM, norgestimate; PASS, post authorization safety study; VTE, venous thromboembolism; WY, women-year. N/A, not applicable.

### A Novel Combined Oral Contraceptive Based on a Native Estrogen, Estetrol

**Figure 6:**
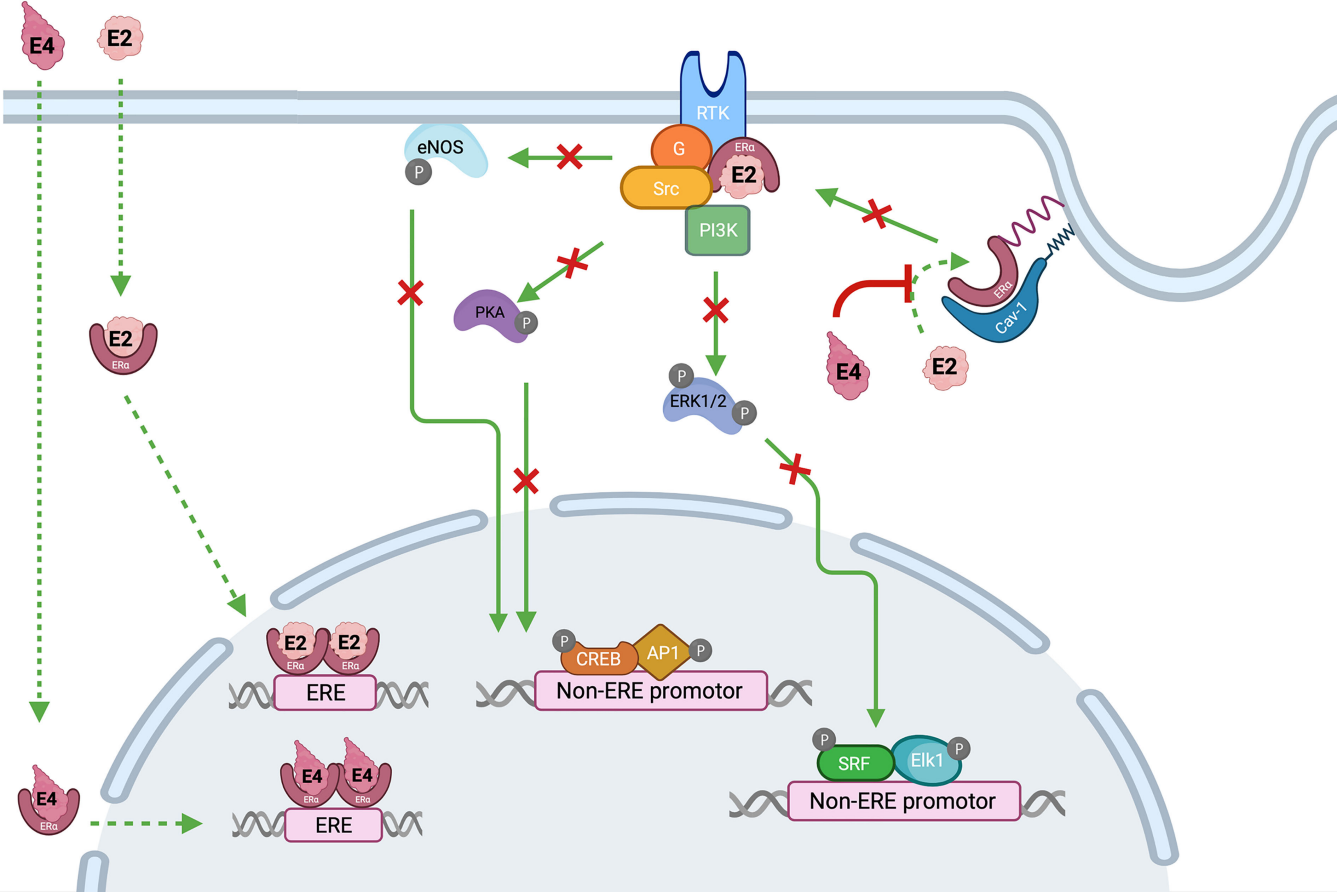
Interaction between estrogens, i.e., estradiol (E2) and estetrol (E4) and estrogen receptors alpha (ERα). Estradiol activates both membrane and nuclear actions of ERα while E4 is an agonist of nuclear activity but an antagonist of the ERα-dependent MISS pathway. Nuclear activity is the result of an interaction between estrogens, i.e., E2 or E4, and the ERα located in the cytoplasm. This binding leads to the dimerization and the translocation of the complex into the nucleus, where it interacts with ERE DNA sequences in target genes. The ERα-dependent MISS pathway consists of a rapid nongenomic activity playing an important role in the endothelial effect of estrogens. Palmitoylation of ERα allows them to anchor to the plasma membrane caveolae where they associate with caveolin-1 (Cav-1). Upon E2 stimulation, ERα is de-palmitoylated and dissociated from Cav-1, to interact with protein kinase (Src and PI3K), G-coupled protein ai (Gai) leading to signaling cascade (Akt, Pka, ERK1/2) and endothelial NO synthase activation. On the other hand, E4 is devoid of ERα MISS activity and even, is also able to antagonize E2-induced MISS effect, especially in the endothelium. AP1, activator protein 1; Cav-1, caveolin 1; CREB, cAMP-response element-binding; Elk1, ETS like-1 protein; ERK, extracellular signal-regulated kinases; eNOS, endothelial nitric oxide synthase; ER, estrogen receptor; ERE, estrogen responsive element; E2, estradiol; E4, estetrol; G, G protein (sub-unit ai); MISS, membrane-initiated steroid signaling; PI3K, phosphoinositide 3-kinase; RTK, receptor tyrosine kinase; SRF, serum response factor.

Estetrol (E4) was discovered in the mid-1960s by Diczfalusy and co-workers by investigating the metabolism of E2 in early pregnancy (87). This steroid molecule, characterized by 4 hydroxyl groups, is exclusively synthetized in the fetal liver which is the only organ capable of both 15α- and 16α-hydroxylation. Estetrol is present in maternal blood and urine from the ninth week of gestation and reaches the maternal circulation through the placenta (88). Produced in increasing quantities during the fetal lifespan, it was suggested that E4 could be a safe estrogenic steroid for human use. Pharmacokinetic studies demonstrated an interesting profile with a good oral bioavailability, no metabolization into active metabolites, i.e. E3, E2 or E1, and a half-life time around 28 hours, suggesting suitability for once daily administration (87). From a pharmacodynamic point of view, E4 showed a high selectivity for the estrogens receptors (ER) and weak interactions with glucocorticoids, progesterone and testosterone receptors (87). The binding affinity for both estrogen α and β receptors (ERα and ERβ) was moderate with a four to five-fold higher affinity for ERα. Like the other estrogens, E4 activates the nuclear ERα but in contrast with other estrogens, it antagonizes the activity of membrane ERα, involved in more rapid signaling pathways. Estrogens can act through this distinctly different pathway by inducing rapid extra-nuclear activity *via* a small pool of ERα located closed to the membrane. This process is defined as membrane-initiated steroid signaling (MISS) and may results in the activation of intracellular signaling pathways (e.g., PI3K, MAPK), the activation of multiple kinases and the production of a variety of downstream second messengers (e.g., nitric oxide, calcium flux, cyclic adenosine monophosphate), directly influencing cell activities that contribute to the regulation of cell survival and proliferation. Interaction of nuclear and MISS pathways remains to date poorly recognized but the kinases activated by the MISS pathway can, in turn, phosphorylate various transcription factors, including ERs and coregulators, and therefore indirectly modulate the transcriptional activity in the nucleus (89–92) (**Figure 6**). Animal and human studies demonstrated that E4 behaves as an agonist in bones, uterus, and brain (hot flush, ovulation inhibition, etc.) through nuclear ERα but as an antagonist of ERα-dependent MISS pathway, and especially in the endothelium (considered as one of the NO synthase activation pathways) (87, 89, 93) With this mode of action, E4 has been recognized as a New Active Substance (NAS) by the European Medicines Agency (EMA) and can be described as the first Native Estrogen with Specific action in Tissues (NEST), a classification which differs from the selective estrogen receptor modulators (SERM) (94). A pharmacological comparison between E4, EE and E2 is shown in **Table 4**.

**Table 4:** Comparison estradiol (E2), ethinylestradiol (EE) and estetrol (E4).

		Estradiol	Ethinylestradiol	Estetrol
**Origin**		Natural Synthetized in the growing ovarian follicle, corpus luteum, placendal, adrenal and Leydig cells, liver, endometrium, brain muscle and fat tissue	Synthetic derivative	Natural Exclusively synthetized in the fetal liver
**Chemical structure**		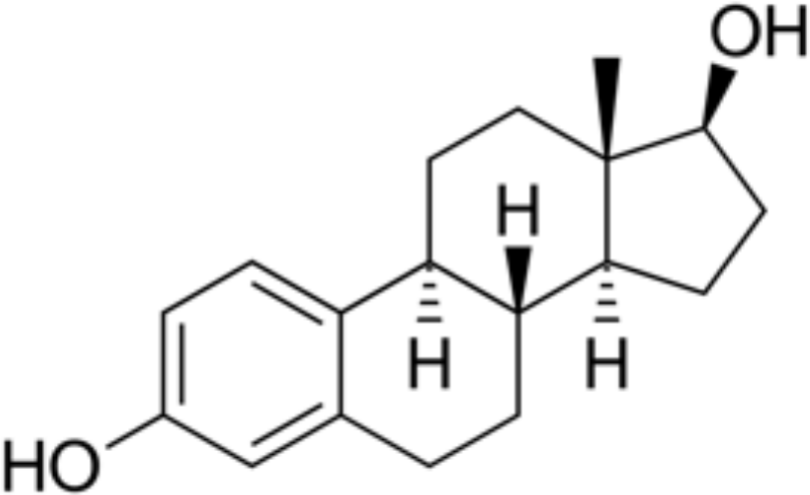	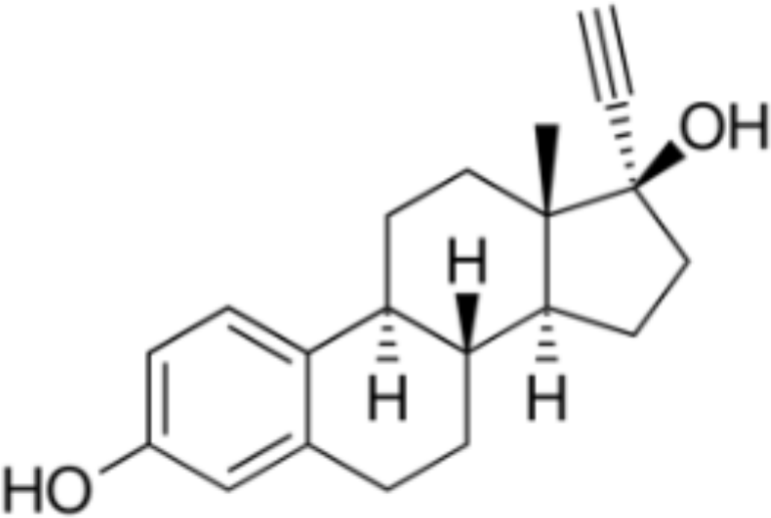	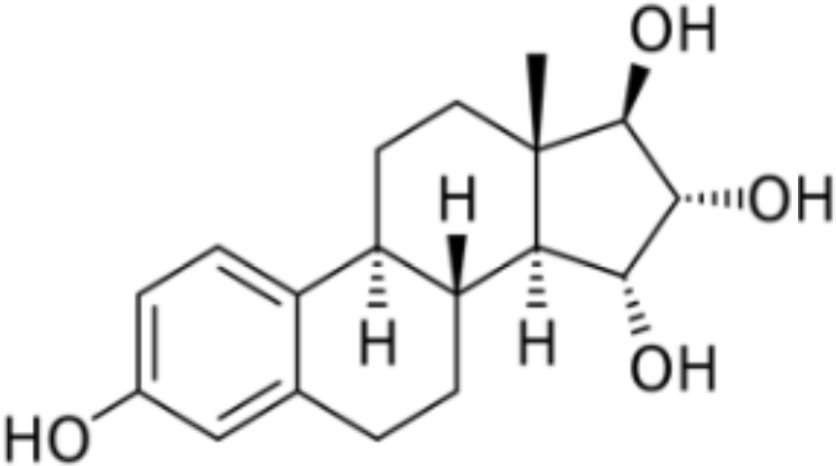
**Dosage in COC**		20-50 µg	1-3 mg	15 mg
**Associated progestogen in COC**		NOMAC, DNG	LNG, NETA, NGM, DSG, GSD, DRSP, CPA, DNG, CMA	DRSP
**PK** **profile**	**Oral bioavailability**	Low oral bioavailability but administered either micronized or esterified.	Good oral bioavailability	Good oral bioavailability
	**Metabolism**	High metabolism into E1 (sulfate) and E3 (sulfate)	High metabolism into various conjugates (glucuronidated, sulfated and hydroxylated metabolites)	No metabolization
	**Half-life time**	Half-life time around 35 hours (E1 serves as precursor of E2 and can be transformed back into E2)	Half-life time around 12 hours	Half-life time around 28 hours
**PD profile**			High selectivity for ER (higher affinity for ERα)	High selectivity for ER (higher affinity for ERα); First Native Estrogen with Specific action in Tissues (NEST)
**Impact on liver protein synthesis**		Minor (non negligeable contribution of E1)	Major	Minor

COC, combined oral contraceptives; CMA, chlormadinone acetate; CPA, cyproterone acetate; DNG, dienogest; DRSP, drospirenone; DSG, desogestrel; ER, estrogen receptor; E1, estrone; E2, estradiol; E3, estriol; GSD, gestodene; LNG, levonorgestrel; NETA, norethisterone acetate; NGM, norgestimate; NOMAC, nomegestrol acetate; PD, pharmacodynamic; PK, pharmacokinetic.

These pharmacological properties made E4 an appropriate candidate for contraception in women of childbearing age or hormonal replacement therapy in menopausal women. Based on the first pre-clinical and clinical data, multiple E4/drospirenone and E4/levonorgestrel dose combinations were investigated and compared to 20 µg of EE in association with 3 mg of drospirenone, for their effects on ovulation inhibition and hemostatic biomarkers (95). Regardless of the dose, i.e., 5 mg, 10 mg or 20 mg of E4 and the progestogen, i.e., either levonorgestrel or drospirenone, E4-containing COCs effectively blocked ovulation and induced minor effects on hemostasis markers (95). In the meantime, an open-label, multi-center, randomized, dose-finding study (FIESTA) was performed to assess bleeding pattern and cycle control of E4 combined with either drospirenone or levonorgestrel (96). The combination of 15 mg E4 with 3 mg drospirenone proved to be the most efficacious with respect to bleeding and cycle control and showed the most satisfaction among the users (96, 97). Further hemostasis investigations revealed that this novel E4-containing COC had a very low impact on the coagulation and fibrinolytic systems (98).

In light of the clinical efficacy and safety data from the Phase II program, the association of E4 at the dose of 15 mg with 3 mg of drospirenone (monophasic 24 + 4 regimen) was selected for phase III development (96). The E4 Female Response concerning Efficacy and safety of Estetrol/Drospirenone as Oral contraceptive in Multicentric study (FREEDOM) Phase III program consisted of two open-label, single arm studies, one performed in Europe and Russia and the other one in US and Canada totaling 3,725 women. The studies confirmed the contraceptive efficacy, a good bleeding profile and cycle control and also reported a high satisfaction rate (99). This led to the approval of E4/drospirenone by several regulatory agencies since the beginning of 2021 (100, 101).

## State-of-the-Art and Perspectives

### The European Medicines Agency Referral: What Needs To Be Done?

In 2013, France informed the EMA, pursuant to Article 31 of Directive 2001/83/EC, of their consideration that the benefit-risk balance of CHCs had become unfavorable in the currently authorized indication due to the increased risk of VTE. The Pharmacovigilance Risk Assessment Committee (PRAC) was then requested to give a recommendation on whether the indication of medicinal products containing chlormadinone acetate, desogestrel, dienogest, drospirenone, etonogestrel, gestodene, norelgestromin, norgestimate, and nomegestrol acetate should be restricted and/or any other regulatory measures taken. In the context of this referral, all pharmacoepidemiology studies on CHCs were reviewed in order to assess the estimated relevant risk of VTE associated with each CHC preparation (**Table 3**). Oral contraceptives considered as the safest in terms of VTE risk were preparations containing EE associated with either levonorgestrel, norethisterone acetate or norgestimate. The highest estimated incidence of VTE was observed with COCs containing EE associated with gestodene, desogestrel or drospirenone (i.e., 9-12 VTE/10,000 women a year) but compared with pregnancy and postpartum period (i.e., 20 VTE/10,000 women a year), it remained lower (84, 85). It was also highlighted that the risk of VTE was higher during the first year of use or following a restart after a one-month period without treatment (84).

As a risk minimization strategy, the PRAC considered that modifications in the summary of product characteristics (SmPC) were required in order to strengthen information relative to the associated risk of VTE. In addition, they recommended the implementation of educational measures to increase healthcare professionals and women awareness regarding the contraindications and the risk factor of VTE and they mentioned that the individual’s risk should be re-evaluated periodically as risk factor for VTE can change over the course of a lifetime (84). On this basis, several guidelines offer support in tailoring contraceptive therapies according to the patient’s profile. There are the US Medical Eligibility Criteria for contraceptive use (102), the World Health Organization Medical Eligibility Criteria for contraceptive use (Fifth Edition, 2015) (103) and the UK Medical Eligibility Criteria for contraceptive use (UKMEC 2016) (104).

### The Challenge of Identifying Women at Higher Risk of VTE, Depending on Their Hormonal Status

Many factors must be considered and discussed with the women when choosing a contraceptive method. In addition to the efficacy, the tolerability and the additional health benefit, the risk of VTE is an important element that must be evaluated. At the present time, this risk is only assessed based on clinical characteristics and does not rely on formal algorithm including phenotypic coagulation screening with laboratory tests. The standard of care, when starting a contraception, consists of identifying the presence of hereditary thrombophilia solely on the basis of familial history of VTE; a strategy which has been reported to be of low sensitivity and predictive value (105, 106). Indeed, identifying a thrombosis within the family does not necessarily mean that there is an underlying thrombophilia. Genetic risk factors alone contribute to only 30% of the family history of VTE (107). Moreover, exclusion of the main hereditary thrombophilia does not mean that a woman will not suffer from thrombotic event under COC use (107). Venous thromboembolism is a multifactorial disease whose occurrence depends on the interaction between gene defects and environmental factors (**Figure 2**) (105). As a result, exposure of high-risk situation such as surgery, trauma, immobilization (e.g., casts, long-range travel/flights), pregnancy or hormonal therapy may trigger a thrombotic event in individuals either in absence or presence of genetic mutations, suggesting that the evaluation should be on the phenotypic rather than the genotypic thrombophilia expression.

There are five well-known genetic thrombophilia that can be divided in two main categories: gain of function mutations and loss of function mutations. Gain of function mutations include prothrombin mutation G20210A and factor V Leiden, which are the more frequent genetic risk factors observed in the Caucasian population. The prevalence reaches 2% for G20210A mutation and 5% for FV Leiden mutation. In contrast, they are much rarer in African and Asian populations (108). The risk of first VTE event is 3- to 7-fold higher in heterozygous carriers while it may reach a relative risk of 30 to 80 in homozygous carriers (109). Mutations, that confer a loss of function, concern deficiencies of protein C, protein S and antithrombin. These mutations are less frequent with a prevalence below 1% but they are associated with a 10- to 50-fold risk of first VTE (109). The presence of one of the above mutations with COCs leads to a synergistic and amplificative (rather than an additive) prothrombotic effect (110). Hugon-Rodin et al. and Khialani et al. investigated the joint effect of COCs and genetic mutation, e.g., FV Leiden mutation or G20210A mutation. Both research groups have calculated a synergy index (SI), reflecting the amplificative effect of the combination of a genetic mutation with COCs above the simple addition of the independent risk alone (110, 111). Based on this, Khialani and co-workers estimated the odds ratios at 19.3 and 24.0 in carriers of FV Leiden mutation and G20210A mutation when using the pill, respectively (110).

Consequently, there is a need of being able to identify the baseline risk of VTE in women before introducing hormonal contraceptives (112). In 2015, Gene Predictis^®^ launched the Pill Protect^®^ on the Swiss market (113). This diagnostic device is based on an algorithm which considers nine polymorphisms and four clinical risk factors associated with VTE development as well as the potential COC that could be prescribed (**Table 5** **)**. To assess the performance of this test, a comparison was performed to the “current” practice, which was based on oral anamnesis of the patient, and to the genotyping for FV Leiden and G20210A mutation.^1^ Results of the ROC curves analyses, reflected by the area under the curve, was higher (i.e., 0.71) with the Pill Protect^®^ compared to the oral anamnesis of the patient (i.e., 0.61) or the simple genotyping of FV Leiden and G20210A mutation (i.e., 0.67). These data revealed the usefulness of this prognostic device and reopened the reflection of performing biological investigations before introduction of a contraceptive method (114). Although this method is promising, the algorithm does not take into account a deficiency of protein S, protein C or antithrombin and in addition, many users of COCs developing VTE do not have a recognized hereditary coagulation problem but show instead a high responsiveness to estrogenic compounds (118). An interesting approach would be to have one or several biomarkers to establish the “coagulability status” of the patient, revealing phenotypic rather than just genotypic particularities.

**Table 5:** Clinical and genetic parameters assessed with the screening test Pill Protect^®^.

Clinical variables	Age	
	BMI	
	Smoking habits	
	Family history of VTE	
**Genetic variables**	Gene: F5 -SNP: rs6025 Allele: A	Gene encoding for coagulation factor V. The resulting re6025(A) allele is known as Factor V Leiden mutation which leads to a resistance to the activated protein C and an increased risk of thrombosis (114).
	Gene: F2 -SNP: rs1799963 Allele: A	Gene encoding for prothrombin. The resulting rs199963(A) allele is known as G20210A mutation which leads to increased plasma prothrombin levels and an increased risk of thrombosis (114).
	Gene: ABO -SNP: rs8176719 Allele: G -SNP: rs8176750 Allele: C	Gene encoding for ABO subtype. The rs8176719(G) and rs8176750(C) alleles encodes for non-O blood groups and are associated with an increased risk of VTE through modifications of von Willebrand Factor (VWF) and factor VIII (FVIII) plasma levels (115).
	Gene: F11 -SNP: rs2289252 Allele: T	Gene encoding for coagulation factor XI. The rs2289252(T) allele is associated with increased FXI activity leading to a procoagulable state (115).
	Gene: CYP2C9 -SNP: rs1799853 Allele: T	Gene encoding for cytochrome CYP2C9 involved in the metabolism of EE. The rs1799853(T) allele could induce a decrease in the metabolism of EE, thus increasing its plasma levels and therefore the global estrogenicity (114).
	Gene: PROCR -SNP: rs9574 Allele: G	Gene encoding for activated protein C receptor involved in the activation of the anticoagulant pathway. The rs9574(G) allele has been reported to be at increased risk of VTE compared to C allele (115).
	Gene: SUGCT -SNP: rs4379368 Allele: T	Gene encoding for the succinate-hydroxymethylglutarate CoA-transferase. The rs4379368(T) allele has been associated with migraine susceptibility. The combination of both migraine and COC could further increase the risk of cardiovascular diseases (116).
	Gene: KNG1 -SNP: rs710446 Allele: C	Gene encoding for kininogen-1. This protein plays an important role in the coagulation process by assembling the kallikrein-kinin system. The rs710446(C) allele has been associated with shortened aPTT levels and an increased risk of VTE (117).

BMI, Body Mass Index; SNP, single nucleotide polymorphism; VTE, venous thromboembolism.

Global sensitive assays like the ETP-based APC resistance assay could be potential candidates. One rationale and affordable perspective could be the screening of the coagulability state in order to provide objective data for the gynecologist to support the prescription of the most appropriate contraceptive method. In case of an abnormal result, which suspects the presence of an underlying pathology, the women can be referred to a hematologist for further investigations. This is of particular importance since the identification of coagulopathies may drive the behavior of both the patient and the healthcare professionals for the entire lifetime of the woman. Subsequently, a second testing after a sequence of one cycle of hormonal treatment would allow identifying women with an abnormal rise of the nAPCsr which may reveal an over-sensitivity to the estrogenic effect of COC or particularities in the metabolism of the concerned COC.

A global screening test would represent a more appropriate and cheaper alternative than a full thrombophilia tests panel for assessing the risk since, this test does not only focus on the inherited coagulopathies but also assesses the individual sensitivity towards COCs. Furthermore, information on the coagulability status could not only reduce the risk of COC-induced thrombosis but also the incidence of thrombotic events in situations associated with elevated thrombotic risk. It has to be reminded that venous thromboembolisms and pulmonary embolisms are associated with a significant mortality but also with a high morbidity rate leading to expense costs for the diagnosis, the treatment and the management of any thrombotic related disability such as recurrent VTE, post thrombotic syndrome or chronic pulmonary hypertension. All of these lead to severe impairment of the affected women, an important financial burden within personal expenses, healthcare resources and societal costs (112). Obviously, such strategies need to be evaluated by proper epidemiological and cost-effectiveness studies but with the advent of new technologies permitting the global assessment of the coagulation process, there is a new era for management of women willing to get the most appropriate and safe contraceptive method based on their individualized profile.

## Conclusion

Over the last 60 years, efforts have been made to reduce the risk of venous thromboembolism events associated with combined oral contraceptives, and today, all strategies seem to be moving towards the safe use of these products. With novel formulations on the market, i.e., estradiol- and estetrol-based combined oral contraceptives, the association of ethinylestradiol with levonorgestrel should no longer be the only option for minimizing the risk of venous thromboembolism associated with combined oral contraceptives use. This has not been discussed in this review but there are obviously alternatives to estroprogestative combinations, e.g., progestin-only pills or intra-uterine devices and as a perspective, a review discussing in details the medical eligibility criteria for contraceptive use as well as the different hormonal methods of contraception, would be of great interest. In addition to the development of safer products, attempts are being made to improve the management of patients who desire to start a contraceptive therapy. The proposal of a global screening test before the initiation of a contraceptive therapy could significantly reduce the 22,000 cases of thrombosis observed each year in Europe following the use of combined oral contraceptives (114).

## Author Contributions

LM and JD made substantial contributions to the literature search and to the writing of the manuscript. HH, J-MD, and UG contributed to the final version of the manuscript. LM achieved the adjustments after the review by other authors and gave final approval of the submitted version of the manuscript. JD was in charge of supervising the project. All authors contributed to the article and approved the submitted version.

## Funding

This project was financed by the Walloon Region in Belgium (government source) – convention 8031.

## Conflict of Interest

UG is senior consultant of Mithra Pharmaceuticals, Liège, Belgium. JD is CEO and founder of QUALIblood s.a. and reports personal fees from Daiichi-Sankyo, Diagnostica Stago, DOASense, Gedeon Richter, Mithra Pharmaceuticals, Norgine, Portola, Roche and Roche Diagnostics.

The remaining authors declare that the research was conducted in the absence of any commercial or financial relationships that could be construed as a potential conflict of interest.

## Publisher’s Note

All claims expressed in this article are solely those of the authors and do not necessarily represent those of their affiliated organizations, or those of the publisher, the editors and the reviewers. Any product that may be evaluated in this article, or claim that may be made by its manufacturer, is not guaranteed or endorsed by the publisher.

## Footnotes

1 It has to be noted that the genotyping of FV Leiden and G20210A mutations is not part of the current practice.
